# Factors Influencing Survival in Stage IV Colorectal Cancer: The Influence of DNA Ploidy

**DOI:** 10.1155/2013/490578

**Published:** 2013-06-04

**Authors:** Ioannis D. Xynos, Nicolaos Kavantzas, Smaro Tsaousi, Michalis Zacharakis, George Agrogiannis, Christos Kosmas, Andreas Lazaris, John Sarantonis, Stavros Sougioultzis, Dimitrios Tzivras, Aris Polyzos, Efstratios S. Patsouris, Nikolas Tsavaris

**Affiliations:** ^1^Medical Oncology, Propedeutic, Laikon General Hospital, Medical School, National and Kapodistrian University of Athens, Mikras Asias 7, 11527 Athens, Greece; ^2^1st Department of Pathology, Propedeutic, Laikon General Hospital, Medical School, National and Kapodistrian University of Athens, Mikras Asias 7, 11527 Athens, Greece; ^3^2nd Department of Medical Oncology, Metaxa Cancer Hospital, Mpotasi 51, 18537 Piraeus, Greece; ^4^Gastroenterology Unit, Department of Pathophysiology, Propedeutic, Laikon General Hospital, Medical School, National and Kapodistrian University of Athens, Mikras Asias 7, 11527 Athens, Greece; ^5^1st Department of Internal Medicine, Propedeutic, Laikon General Hospital, Medical School, National and Kapodistrian University of Athens, Mikras Asias 7, 11527 Athens, Greece

## Abstract

*Objective*. To evaluate the prognostic significance of microscopically assessed DNA ploidy and other clinical and laboratory parameters in stage IV colorectal cancer (CRC). *Methods*. 541 patients with histologically proven stage IV CRC treated with palliative chemotherapy at our institution were included in this retrospective analysis, and 9 variables (gender, age, performance status, carcinoembryonic antigen, cancer antigen 19-9, C-Reactive Protein (CRP), anaemia, hypoalbuminaemia, and ploidy (DNA Index)) were assessed for their potential relationship to survival. *Results*. Mean survival time was 12.8 months (95% confidence interval (CI) 12.0–13.5). Multivariate analysis revealed that DNA indexes of 2.2–3.6 and >3.6 were associated with 2.94 and 4.98 times higher probability of death, respectively, compared to DNA index <2.2. CRP levels of >15 mg/dL and 5–15 mg/dL were associated with 2.52 and 1.72 times higher risk of death, respectively. Hazard ratios ranged from 1.29 in patients mild anaemia (Hb 12–13.5 g/dL) to 1.88 in patients with severe anaemia (Hb < 8.5 g/dL). Similarly, the presence of hypoalbuminaemia (albumin < 5 g/dL) was found to confer 1.41 times inferior survival capability. *Conclusions*. Our findings suggest that patients with stage IV CRC with low ploidy score and CRP levels, absent or mild anaemia, and normal albumin levels might derive greatest benefit from palliative chemotherapy.

## 1. Introduction

More than 1 million individuals worldwide will be diagnosed with colorectal cancer (CRC) every year [1, 2]. Approximately 35% of CRC patients present with stage IV metastatic disease at the time of diagnosis, and 20%–50% with stage II or III disease will progress to stage IV at some point during the course of their disease [3–5]. Stage IV CRC carries an unfavourable outcome as the 5-year survival rate is less than 10% [4, 5] with a median survival time of about 6–12 months [6, 7].

In metastatic CRC, surgery and/or chemotherapy are used mainly with palliative intent. However, as treatment modalities in stage IV CRC are associated with significant complications and increased costs, there is a need to identify prognostic factors which may determine treatment response and survival. It is anticipated that such an approach could refine palliative management according to the likelihood of clinical benefit [8]. 

As part of our systematic search for prognostic factors in CRC, this study expanded our previous work [9] by evaluating the prognostic significance of DNA ploidy in addition to other clinicopathological factors in a cohort of patients with stage IV metastatic disease receiving palliative chemotherapy. 

## 2. Materials and Methods

### 2.1. Patients and Data Sources

The population under study has been described thoroughly in a previous report [9]. Briefly, the medical records of 541 patients with histologically proven CRC (UICC stage IV) between 1998 and 2008 were retrospectively reviewed. All were consecutive nonselected cases from a single centre and all patients were treated outside of clinical trials. No patients were candidates for surgical treatment (either curative or palliative); however, all received palliative chemotherapy according to established protocols. Chemotherapy regimens were based on single agent leucovorin modulated 5-FU (Mayo clinic or AIO regimens) or combination treatments of 5-FU (DeGrammont or simple infusion and leucovorin) with either oxaliplatin or irinotecan, or capecitabine with or without bevacizumab or cetuximab. Records with complete data (for the parameters used as prognostic factors) were included in the analysis. Followup was continued until death from CRC or from any other cause, and patients who remained alive were censored as of January 1, 2009. Overall survival was the primary endpoint. This protocol has been approved by the National and Kapodistrian University of Athens ethics committee.

### 2.2. Prognostic Variables

Nine possible prognostic variables were selected. These included patient-related variables such as age (≤60 years or >60 years), gender, and performance status (PS) according to the Karnofsky Performance Status Scale Index. For the evaluation of continuous laboratory parameters, we used group categorisations: for haemoglobin: >13.5 g/dL, 12–13.5 g/dL, 10–12 g/dL, 8.5–10 g/dL, and <8.5 g/dL; for carcinoembryonic antigen (CEA): normal ≤5 mg/dL and elevated >5 mg/dL; for cancer antigen 19-9 (CA 19-9): normal ≤30 U/L and elevated >30 U/L; for C-reactive protein (CRP): normal <5 mg/dL, moderately elevated 5–15 mg/dL, and highly elevated >15 mg/dL; and for albumin: normal >5 gr/dL and low ≤5 gr/dL. For ploidy score (DNA index), group categorisation was also applied for analytical purposes: <2.2, 2.2–2.6, >3.6.

### 2.3. DNA Measurements (Ploidy)

For DNA measurements, the Feulgen staining technique was applied as previously described [10]. The nuclei of Feulgen-stained cells were evaluated for DNA ploidy using a Nikon eclipse microscope (Nikon, Japan) connected with a Nikon CCD videocamera and an IBM Pentium 4/PC cell measurement software (Image Pro Plus v. 5.1, Media Cybernetics Inc, Silver Springs, MD, USA). Areas of the Feulgen-stained sections containing pathological lesions, identified in adjacent H&E stained slides, were selected for DNA content analysis. A total of 200–300 nuclei with clear boundaries appearing to have no loss of membrane integrity were analyzed in each tissue sample. Cytometry measurements were performed with a magnification of ×200 and calculated automatically according to the algorithms described previously by measuring the nuclear integrated optical density (IOD), representing the cytometrical equivalent of DNA content [11]. The procedure was performed for all nuclei, and the overall mean represents DNA content or DNA index (DI). Mean IOD of human lymphocytes (control cells) was used as the diploid standard (2c) and reference for DI calculation for targeted cells. DNA histograms were generated and a tumour was classified as diploid if the DI ranged from 0.9 to 1.1 and the relevant DNA histogram revealed only 1 peak at 2c and aneuploid if any from the previous 2 criteria was absent.

### 2.4. Statistical Analysis

Descriptive statistics were calculated with the measures of means, medians, and standard deviation for quantitative parameters and counts/percentages for discrete factors. Overall survival was studied with the use of Kaplan-Meier method. Survival differences between groups are studied with the use of log-rank test. A multivariate Cox-regression model was implemented to study the simultaneous effect of parameters on survival after taking into account the parallel effect of remaining factors. Best model selection was based on manual and automated forward techniques. Results of regression analyses were displayed in the form of regression estimates tables. Hazard ratios of outcomes under study were calculated for each parameter estimate as well as 95% confidence intervals. Categorical covariates were compared with a predefined reference category. All analyses were performed at a significance level of *α* = 0.05 with the use of the statistical package SPSS 12.0.

## 3. Results and Discussion

### 3.1. Results

#### 3.1.1. Patients

A total of 541 patients were included in the study, with median age of 61.00 years, a mean age of 60.33 years, and standard deviation of 7.35 years. The frequencies of the clinical variables are shown in Table 1.

#### 3.1.2. Survival Analysis

**Figure 1:**
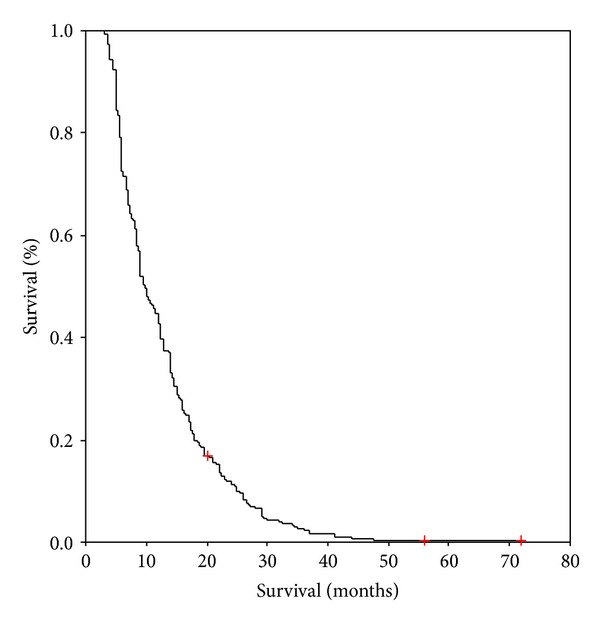
Overall survival (*n* = 541).

Survival data were collected for all patients. Based on the Kaplan-Meier method, the mean survival time was recorded at 12.8 months (95% confidence interval (CI) 12.0–13.5 months), with a median survival of 9.8 months (95% CI 8.8–10.8 months) (Figure 1).

#### 3.1.3. Univariate Analysis

In the univariate analysis, CRP, Hb, albumin, and ploidy scores were related to survival outcome at a significance level of *P* < 0.001.

### 3.2. Multivariate Analysis

Factors found to have strongest significance of a relation to survival according to the bivariate analysis were entered into the multivariate analysis model. Factors were added and excluded using the change in likelihood between models as inclusion and exclusion criteria. Forward automated procedures resulted in the final model, which is described in Table 2. 

### 3.3. Hazard Ratios of Risk Factors

**Figure 2:**
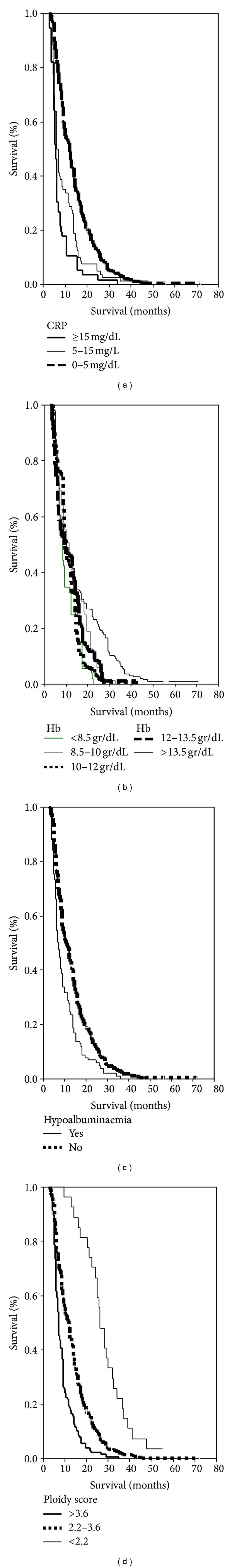
Survival data according to CRP (a), anaemia (b), hypoalbuminaemia (c), and DNA ploidy (d).

Probability of death increased with increased CRP at presentation; patients with CRP > 15 mg/dL had 2.52 higher risk of death and patients with CRP 5–15 mg/dL had 1.72 times higher risk of death than patients with CRP < 5 mg/dL (Figure 2(a)). Anaemia was also associated with an adverse outcome. In particular HRs ranged from 1.29 in patients who presented with mild anaemia (Hb 12–13.5 g/dL) to 1.88 in patients with severe anaemia (Hb < 8.5 g/dL) (Figure 2(b)). Similarly, patients with low albumin levels (<5 g/dL, hypoalbuminaemia) had 1.41 times higher probability of death than did those with normal albumin levels (Figure 2(c)). Finally, a high ploidy score was associated with worst survival prognosis as patients with ploidy scores 2.2–3.6 or >3.6 had 2.94 and 4.98 times higher probability of death, respectively, as compared to those patients with ploidy score <2.2 (Figure 2(d)). 

## 4. Discussion 

This pooled analysis based on the individual data of 541 stage IV colorectal cancer patients treated with palliative chemotherapy confirms the prognostic value of previously identified factors such as CRP, Hb, and albumin and strengthens the existing data from other studies supporting the prognostic significance of DNA ploidy in stage IV colorectal cancer.

CRP is synthesized by the liver and is a nonspecific but sensitive marker of inflammation. Its production is induced by proinflammatory cytokines such as Interleukin-6 (IL-6), IL-8, and tumour necrosis factor alpha (TNF-*α*) and its levels have been positively correlated with weight loss, anorexia-cachexia syndrome, extent of disease, and recurrence in many cancer types including CRC [12]. Preoperatively elevated serum CRP levels have been shown to be associated with increased incidences of liver metastases, peritoneal carcinomatosis, histopathologic lymph nodes metastasis, intravascular invasion, and detrimental 1-, 2- and 3-year survival rates in CRC [13], and these results have been supported by others [14]. Although there appears to be no difference in Dukes' stage between patients with normal or increased preoperative CRP levels [15], CRP has been shown to specifically influence survival in patients with Dukes' C and D tumours. In the advanced disease setting in particular, a recent analysis of a homogeneous cohort consisting of 50 patients with peritoneal carcinomatosis has demonstrated an association between elevated plasma CRP levels at the time of diagnosis and overall survival [16]. On the opposite end, Chung and Chang have advocated on the lack of prognostic significance of CRP in CRC by a multivariate analysis of rather small heterogeneous cohort consisting of 172 patients with CRC at various stages [17]. 

The association between anaemia and inferior survival capability has been widely validated in previous studies including a multivariate analysis of 3.825 patients with stage IV CRC treated with palliative 5FU-based chemotherapy in the setting of 22 multinational trials by Köhne et al. [18]. Serum albumin reflects the nutritional status of patients depicting general condition, including reserve capacity, and its predictive value on metastatic CRC outcome has been well documented [18–23]. 

The prognostic significance of DNA content (DNA ploidy or index) in CRC has been extensively investigated in the past with controversial results. The majority of the reported studies have employed flow cytometric derived DNA ploidy, and the American Society of Clinical Oncology Tumour Markers Expert Panel has reviewed fifteen articles (encompassing 14 independent series) evaluating the prognostic role of flow cytometric derived ploidy in CRC to support its recommendation regarding the unsuitability of this marker as a prognostic determinant due to largely controversial results [24]. To some extent, disparate results in DNA ploidy studies have been ascribed to the differing techniques employed (flow cytometry versus DNA image cytometry) and the heterogeneity in the nuclear DNA content in colonic tumour cells; hence, image cytometry has generally been considered superior to flow cytometry as only tumor cells are used for DNA measurement [25]. Despite its technical advantages, image cytometry has only been applied to a limited number of studies which were particularly aimed to identify patients with stage II disease with high risk of recurrence following curative resection and assess the survival benefit of adjuvant chemotherapy. One of these earlier studies by Nori et al. [26] demonstrated that aneuploidy was associated with significantly higher tumour recurrence rate (*P* = 0.024) and shorter overall survival (*P* < 0.002) but was hampered by small patient number (*n* = 20). Subsequent studies by Kay et al. [27] and Buhmeida et al. [28] in larger patient cohorts (*n* = 168 and *n* = 253, resp.) demonstrated the prognostic significance of DNA image cytometry in stages II CRC and have evolved this marker as a major determinant for administering adjuvant chemotherapy in stage II disease. These results were reiterated by a meta-analysis of 63 studies reporting outcome in 10126 patients, 60.0% of whom had chromosomal instability positive (CIN+, i.e., aneuploid/polyploid) tumours whereby it was shown that patients with CIN+ CRC and stages II-III disease appear to have a poorer survival in terms of overall survival and progression free survival irrespective of whether these receive adjuvant therapy. In stage IV disease, the data were inconclusive due to low patient numbers confounded by high degree of heterogeneity [29]. 

The limitations of our study centre mainly on the retrospective nature of the analysis and the objectivity of the methodology applied to assess DNA ploidy. Despite these limitations, the study has clinical significance as it validates the usefulness of a number of factors to assess the likelihood of clinical benefit of palliative chemotherapy in stage IV CRC. Clearly, however, these results need to be evaluated in a prospective manner.

## 5. Conclusions

The present study represents a comprehensive analysis of the prognostic significance of a number of factors in a large cohort of stage IV unoperable colorectal cancer patients receiving palliative chemotherapy. Our analysis demonstrated that DNA ploidy, along with simple haematological and biochemical parameters such as Hb, CRP, and albumin, carries the most significant independent effect on the outcome of stage IV CRC. 

## Figures and Tables

**Table 1:** Demographic, clinical, and laboratory variables in the study population (*n* = 541).

Factor	*n*	%
Gender		
Males	298	55.1
Females	243	44.9
Age		
≤60 years	261	48.2
>60 years	280	51.8
Pretreatment PS		
70	75	13.9
80	160	29.6
90	155	28.7
100	151	27.9
CEA		
≤5 mg/dL	134	24.8
>5 mg/dL	407	75.2
CA 19-9		
≤30	152	28.1
>30	389	71.9
CRP		
<5 mg/dL	405	74.9
5–15 mg/dL	80	14.8
>15 mg/dL	56	10.4
Anaemia		
Hb > 13.5 g/dL	198	36.6
Hb 12–13.5 g/dL	94	17.4
Hb 10–12 g/dL	112	20.7
Hb 8.5–10 g/dL	85	15.7
Hb < 8.5 g/dL	52	9.6
Hypoalbuminaemia		
No	440	81.3
Yes	101	18.7
Ploidy		
<2.2	27	5.1
2.2–3.6	375	70.8
>3.6	128	24.2

PS: performance status; CA 19-9: cancer antigen 19-9; CEA: carcinoembryonic antigen; CRP: C-reactive protein; Hb: Hemoglobin.

**Table 2:** Final Cox proportional odds regression model.

Variable	*B*	SE	Wald	*P*	Hazard ratio	95.0% CI for Exp(*B*)	
						Lower	Upper
CRP 5–15 mg/dL versus <5 mg/dL	0.552	0.131	17.652	0.000	1.737	1.343	2.248
CRP > 15 mg/dLversus <5 mg/dL	0.924	0.153	36.303	0.000	2.520	1.866	3.404

Hb 12–13.5 g/dL versus >13.5 g/dL	0.259	0.133	3.780	0.052	1.295	0.998	1.681
Hb 10–12 g/dL versus >13.5 g/dL	0.471	0.128	13.543	0.000	1.602	1.246	2.059
Hb 8.5–10 g/dL versus >13.5 g/dL	0.407	0.137	8.843	0.003	1.503	1.149	1.965
Hb < 8.5 g/dL versus >13.5 g/dL	0.636	0.168	14.406	0.000	1.889	1.360	2.622

Hypoalbuminaemia (Yes versus No)	0.347	0.116	8.994	0.003	1.415	1.128	1.776

Ploidy score 2.2–3.6 versus <2.2	1.081	0.209	26.740	0.000	2.947	1.957	4.439

Ploidy score >3.6 versus <2.2	1.606	0.225	50.780	0.000	4.982	3.203	7.748

SE: standard error, CI: confidence interval; CRP: C-reactive protein; Hb: hemoglobin.
